# Patient satisfaction at accredited antiretroviral treatment sites in the Gert Sibande District

**DOI:** 10.4102/phcfm.v6i1.627

**Published:** 2014-11-27

**Authors:** Selente Bezuidenhout, Damilola A. Ogunsanwo, Elvera A. Helberg

**Affiliations:** 1 Department of Pharmacy, University of Limpopo, Medunsa Campus, South Africa

## Abstract

**Background:**

Patient satisfaction has been used as a significant indicator of quality services provided by healthcare personnel. With the largest antiretroviral therapy (ART) programme in the world, the healthcare industry is struggling increasingly with challenges of meeting patients’ requirements and expectations for quality ART service provision. This study was conducted in order to identify the importance of factors contributing to satisfaction or dissatisfaction.

**Aim:**

This study sought to explore and describe the general satisfaction or dissatisfaction of patients with accredited ART hospital sites at public health facilities in the Gert Sibande District, Mpumalanga and to identify factors contributing to either satisfaction or dissatisfaction.

**Setting:**

Six hospitals that initiated ART in the district, participated in the study.

**Method:**

The study was conducted using a sample of 300 patients. Proportional random sampling was used in selecting the number of patients from each facility. A structured interview with each participating patient was conducted using a standardised structured questionnaire. The first available required number of patients that complied with requirements from each of the six hospitals was selected for the interview. Descriptive statistics were used to analyse data and data with qualitative aspects were captured and categorised manually.

**Results:**

The major factors contributing to satisfaction included the availability of medicines and knowledge regarding how to take medication. Factors contributing to dissatisfaction on the part of the patients included confidentiality issues, long waiting periods, shortage of staff and dirty toilets.

**Conclusion:**

This study indicated general satisfaction with the ART-related services at the accredited ART hospital sites in the Gert Sibande District. Regular monitoring and evaluation are recommended.

## Abstract (fr)

**Satisfaction des patients aux centres de traitement antiviral accrédités dans le District Gert Sibande, dans la Province du Mpumalanga.**

**Contexte:**

La satisfaction des patients a été utilisée comme indicateur principal des services de qualité donnés par le personnel soignant. Avec le programme le plus vaste de thérapie antirétrovirale (ART) du monde, le secteur médical se trouve confronté de plus en plus aux défis de satisfaire les besoins et les attentes des patients demandant des prestations de service ART de qualité. Cette étude a été menée afin d’identifier l’importance des facteurs contribuant à la satisfaction ou au mécontentement des patients.

**Objectifs:**

Cette étude s’est efforcée d’étudier et de décrire la satisfaction ou le mécontentement général des patients dans les services de santé publique avec des services ART accrédités dans le District Gert Sibande au Mpumalanga et d’identifier les facteurs qui contribuent à la satisfaction ou au mécontentement des patients.

**Lieu:**

Six hôpitaux qui ont mis en place ART dans le district, ont participé à l’enquête.

**Méthode:**

L’enquête s’est faite sur un échantillon de 300 patients. On a utilisé l’échantillonnage aléatoire proportionnel pour sélectionner le nombre de patients de chaque endroit. On a eu une entrevue structurée avec chaque patient participant à l’aide d’un questionnaire normalisé et structuré. On a sélectionné pour les entrevues le nombre requis de patients conformes aux exigences dans chacun des six hôpitaux. On a utilise les statistiques descriptives pour analyser les données, puis on a saisi les données aux aspects qualitatifs et on les a classées manuellement par catégories.

**Résultats:**

Les facteurs principaux contribuant à la satisfaction des patients comprenaient la disponibilité des médicaments et les connaissances de base sur la façon de les utiliser. Les facteurs contribuant au mécontentement des patients comprenaient les questions de confidentialité, les longues attentes, la pénurie de personnel et les toilettes sales.

**Conclusion:**

Cette étude a montré une satisfaction générale envers les services ART aux hôpitaux accrédités d’ART dans le district Gert Sibande. Il est recommandé de faire des évaluations et des contrôles réguliers.

## Introduction

The Operational Plan for Comprehensive Human Immunodeficiency Virus (HIV) and Acquired Immune Deficiency Syndrome (AIDS) Care, Management and Treatment (CCMT), was approved by the South African government in 2003. This plan provided for free antiretroviral treatment (ART) at public health facilities.^1^ According to the 2013 Global Update on HIV Treatment, more than 2.1 million people were receiving ART in South Africa.^2^ This high number of patients who qualify for ART increases the workload of the public healthcare system.^3^

Patient satisfaction has become an important performance and outcome measure of healthcare. In a debilitated healthcare system, it is important to ensure quality of care and patient satisfaction in order to make the best use of limited resources.^4,5^ The provision of ART using the public health system can transform HIV infection from a fast, deadly disease into a manageable chronic illness.^6^ However, in resource-limited settings, there are many challenges to successfully scaling-up ART and reorienting service delivery toward chronic disease care. Patient satisfaction surveys can be of benefit to health professionals with regard to identifying areas where service delivery and health expenditure can be improved.^7^ South Africa has the largest ART programme in the world and, as ART treatment access is improving, public health benefits are also increased. Six-month mortality amongst patients at a HIV treatment centre in the Western Cape Province was reduced from 12.7% to 6.6% between 2003 and 2005.^8^

Mpumalanga has the second-highest prevalence of HIV in South Africa. It is estimated that about 10% of the overall population in the Mpumalanga Province lives with HIV. The Gert Sibande District has an HIV prevalence of 40.5%, which is the highest in the Mpumalanga Province. By the end of November 2011, 32 979 patients were receiving ART in the Gert Sibande District.^9^

All Primary Health Care (PHC) clinics and Community Health Centres (CHCs) in the District have been prepared for ART readiness by the provincial government since April 2010.

Although previous studies conducted in the Free State^10^ and KwaZulu-Natal^11^ showed high patient satisfaction with ART-accredited sites, the aim of this study was to determine patient satisfaction with ART-accredited hospital-based sites in the Gert Sibande District, Mpumalanga Province and to identify the factors contributing to either satisfaction or dissatisfaction. This was important in order to define service areas that required improvement and to assist hospital management with regard to targeting interventions for quality improvement.

## Research methods and design

### Study design

The study adopted a quantitative approach using a cross-sectional descriptive design, where data were collected in the form of researcher-administered questionnaires.

### Study sites

The study was conducted at all five district hospitals (Embhuleni, Piet Retief, Carolina, Bethal and Standerton) and at one regional hospital (Ermelo) in the Gert Sibande District.

### Study population

The target population included all patients on ART who attended the six hospitals.

### Sample selection

According to 2011 hospital statistics supplied by the different hospital administration departments, a total number of 695 patients on ART attended these six hospitals on a daily basis. Sample size estimation was performed using nQueryAdvisor, Release 6.0 (Elashoff 2005). The required sample size for 695 patients was at least 248. A sample of 300 patients was studied. With a sample size of 300, a two-sided 95% confidence interval for the proportion of satisfied subjects, using the normal approximation, extended a distance of 0.05 from the observed proportion (calculated from the sample). The expected proportion of satisfied subjects was in the range of 0.65–0.75 (65% – 75%). Proportional random sampling was used to determine the number of patients to be included in the study from each hospital site according to the number of patients attending each hospital site daily. Convenience sampling was used further in order to recruit the required number of patients from each hospital site.

### Inclusion criteria

Patients over 18 years who had received ART for at least four months or longer and were collecting repeat medication were included in the study. The first available required number of patients (sample of convenience) that complied with the criteria from each of the six hospitals, who were willing to participate and who provided consent, completed the questionnaire.

### Data collection

An interview with each participating patient was conducted by using a standardised structured questionnaire. The data collection tool was adapted from a patient exit interview questionnaire that the Pharmacy Department uses and updates every year in order to collect data from PHC Clinics as part of the Research Module in the BPharm curriculum.^12^ However, validity of this questionnaire was again tested during a pilot study that was conducted before the actual research data collection, just to be sure that all questions asked were understood correctly and that the trained data collectors were satisfied with the responses to the questions. The method of data collection from participating patients was selected because of the possibility of differences in patients’ literacy levels. The questionnaire comprised sections on sociodemographics, general facility and healthcare provision, communication and medicine management.

The required number of patients was recruited whilst they were waiting for medication at the pharmacy waiting area. Information leaflets were distributed in the waiting area and the interview took place in the pharmacy’s private consulting room prior to the patients receiving their medication. The study was explained to the patients and consent forms were provided. Patient questionnaires, information leaflets and consent forms were translated into siSwati (translation–back-translation process). The questionnaire was administered in English. A data collector was trained in data collection and interviewing and administered a translated version in siSwati (the main language spoken in Gert Sibande) to the patients when necessary. The sequence of events was practised so as to ensure that all questionnaires were administered in the same way. All responses in siSwati were translated to English through the translation-back-translation process. All the included patients participated both voluntarily and anonymously.

### Data analysis

The data that were collected during the study were captured in a Microsoft Excel^®^ 2010 spreadsheet. Data capturing was verified and validity checks were performed. Data were then analysed using descriptive statistics. Responses to categorical variables were summarised as frequency counts and percentages. All statistical procedures were performed on the Statistical Analysis System (SAS), Release 9.2 or higher (SAS Institute Inc. 2008), running on Microsoft Windows Vista^®^. Responses to open-ended questions were captured and categorised, which allowed for the counting of responses where appropriate.

### Ethical considerations

Ethical approval was obtained from the Medunsa Research and Ethics Committee (MREC/H/14/2011: PG). Approval was also obtained from the Chief Executive Officers at each of the different hospitals, the District Manager of the Gert Sibande District and the Mpumalanga Ethics and Research Committee.

Informed consent was obtained from the patients before participation in the study and the patients were informed that they may withdraw from the study without negative consequences. Interviews were conducted in a private consultation area in order to ensure privacy, safety and confidentiality. The questionnaires were anonymous – the identity of patients was not disclosed and patients were informed of this fact. Data were handled with confidentiality and controlled access to data forms was ensured by keeping the completed questionnaires locked away.

## Results

The majority of the patients (*n* = 165; 55%) fell within the 31–45 year age category. Gender distribution showed that there were 202 women on treatment (69%) compared with 91 (31%) men. The patients had an education level ranging from Grades 8 to 12 and the majority of patients (*n* = 244; 82%) could both read and write. However, 61% (*n* = 181) were unemployed (Table 1).

**TABLE 1:** Socio-demographic characteristics of patients.

Characteristics	Frequency	Percentage
Age (n = 299)*		
18–30	90	30.1
31–45	165	55.2
46–60	42	14.0
61 +	2	0.7
Gender (n = 293)*	
Male	91	31.1
Female	202	68.9
Education level (n = 299)*	
Below Grade 7	81	27.1
Grades 8–12	208	69.6
Tertiary education	10	3.3
Literacy level (n = 298)*	
Cannot read or write	42	14.1
Only read	4	1.3
Only write	8	2.7
Read and write	244	81.9
Socio-economic status (n = 296)*	
Employed	115	38.9
Unemployed	181	61.1
Patients with a disability (physical, intellectual and/or mental) (n = 298)*	
Yes	24	8.1
No	274	91.9

*, *n* < 300 – not all patients answered the specific question.

Of the total number of patients interviewed, 169 (56%) reported not seeing the same healthcare provider whilst 131 (44%) consulted with the same healthcare provider each time. Of all the patients interviewed, 290 (97%) reported that they had privacy during their clinic consultation and the majority (*n* = 293; 98%) of patients also reported that their medical records were kept confidential. A high number of the patients surveyed (*n* = 284; 95%) found their ART site to be convenient to them and 258 (86%) of patients found the waiting area suitable for them whilst waiting for their appointment (Table 2).

**TABLE 2:** General facility and healthcare provision.

Characteristics	Frequency	Percentage
Attended by the same healthcare provider each time (n = 300)	
Yes	131	43.7
No	169	56.3
Privacy during examination (n = 300)	
Yes	290	96.7
No	10	3.3
Confidentiality of medical records (n = 300)	
Yes	293	97.7
No	7	2.3
Convenience of ART hospital site (n = 300)	
Yes	284	94.7
No	16	5.3
Attendance to patients on a first come, first help basis (n = 300)	
Yes	268	89
No	32	11
Suitability of waiting area (n = 299)*	
Yes	258	86.3
No	41	13.7

ART, antiretroviral therapy.

*, *n* < 300 – not all patients answered the specific question.

The majority of the patients (*n* = 289; 96%) reported that their healthcare provider spoke their language. Most of the patients interviewed (*n* = 293; 98%) agreed that their healthcare provider listened to their problems.Table 3 shows that 93% (*n* = 279) of the patients had an opportunity to ask their healthcare provider questions and 98% (*n* = 292) of the patients were satisfied with the explanations provided.Table 3 also indicates that 293 (98%) of the patients reported that the healthcare provider who attended to them was polite and 294 (98%) of the patients were asked to return to the hospital site at a specific time.

**TABLE 3:** Communication.

Characteristics	Frequency	Percentage
Healthcare provider speak your language (n = 300)	
Yes	289	96.3
No	11	3.7
Healthcare provider listens to problems (n = 300)	
Yes	293	97.7
No	7	2.3
Healthcare provider gives opportunity to patients to ask questions (n = 299)*	
Yes	279	93.3
No	20	6.7
Patient satisfaction with answers by healthcare provider (n = 299)*	
Yes	292	97.7
No	7	2.3
Politeness of healthcare provider (n = 300)	
Yes	293	97.7
No	7	2.3
Explanation of CD4 + count to patients (n = 300)	
Yes	276	92.0
No	24	8.0
Healthcare provider asks patient to return to hospital site at a specific time (n = 300)	
Yes	294	98
No	6	2

*, *n* < 300 – not all patients answered the specific question.

Of the patients surveyed, 297 (99%) reported that medicines are always available to them every time they visit the hospital for their repeat treatment. The majority (*n* = 252; 85%) of the patients collected their tuberculosis (TB) and/or other chronic medication on the same day as their visit to the ART clinic. All the patients across the ART facilities indicated that the pharmacist was the dispenser of their medicines during consultation and the majority (*n* = 294; 99%) of the patients understood the directions on the labels of the medicines.Table 4 also indicates that 244 (81%) of the patients preferred to collect their medication at the main pharmacy and 292 (97%) found their medicines easy to administer.

**TABLE 4:** Medicine management.

Characteristics	Frequency	Percentage
Patients receiving medication on time (n = 300)	
Yes	297	99
No	3	1
Collection of tuberculosis and/or chronic medicines at the same time (n = 297)*	
Yes	252	84.8
No	45	15.2
Person dispensing medicines (n = 298)*	
Pharmacist	298	100
Other	0	0
Clear directions on label (n = 298)*	
Yes	294	98.7
No	4	1.3
Knowledge regarding the administration of medication (n = 300)	
Yes	292	97
No	8	3
Collection of medication from the main pharmacy preferred (n = 300)	
Yes	244	81.3
No	56	18.7

*, *n* < 300 – not all patients answered the specific question.

**TABLE 5:** Satisfaction or dissatisfaction with care.

Characteristics	Frequency	Percentage
Would you come to this facility again? (n = 299)*	
Yes	283	94.6
No	16	5.3
Transport to facility difficult (n = 297)*	
Yes	232	78.1
No	65	21.9
Patients who want to be transferred to other institutions (n = 296)*	
Yes	180	60.8
No	116	39.2
General satisfaction or dissatisfaction with care at facility (n = 297)*	
Satisfied	290	97.6
Dissatisfied	7	2.4

*, *n* < 300 – not all patients answered the specific question.

Over all, reflections from the patients interviewed on satisfaction of care in the facilities showed that the majority (*n* = 297; 98%) of the patients were satisfied with the care they receive from the ART sites and 95% (*n* = 283) said that they would come again. However, 180 (61%) of the patients would like to be referred to institutions closer to them and 232 (78%) found transport to be difficult.

## Discussion

Patient satisfaction is one of the vital components for the success of any healthcare service, especially in ART units which play a vital role in the lives of thousands of HIV patients.

The majority of patients were between the ages of 31 and 45, which represents a large percentage of the active South African workforce.^13^ This emphasises the importance of ensuring adequate ART coverage in this population in order to maintain South Africa’s economic integrity. Almost 69% of the patients interviewed were women, which is consistent with previous studies showing that more women than men are receiving ART. Biologically, women are twice more likely to become infected with HIV through unprotected heterosexual intercourse than men and therefore more women present at treatment centres.^14^ In addition, a review of the literature has indicated that men attend ART clinics less frequently than women and they are generally initiated on ART later in the course of their infection. However, the main causes for this gender gap have not yet been explained in full.^15^

The educational level of patients revealed that 70% had between Grade 8 and Grade 12 formal educational levels. Almost 82% of patients could read and write. This high literacy level may be a result of the fact that about 70% of patients had formal education. A high rate of unemployment (61%) was also found amongst the patients and this is more than double the average unemployment rate for South Africa (25%) as revealed by the Statistics South Africa Report in 2011.^16^ However, reasons for unemployment, whether it was the disease or a disability grant or something else, were not investigated.

### General facility and healthcare provision

Less than half of the patients were seen by the same healthcare provider each time they visited the ART hospital sites. This might be because, in most cases, human resource shortages are experienced at the ART hospital sites as there are no specific doctors dedicated to the ART sites in some facilities. However, all the patients interviewed at Carolina Hospital (100%) *did* consult with the same healthcare provider, who was specifically employed for the ART site by a non-governmental organisation (NGO) partnering with government.

Privacy during medical examination and confidentiality of medical records are factors that are of utmost importance to patients. Table 2 showed that 97% of the patients indicated privacy during medical examinations and 98% of patients perceived that their medical records were kept confidential. Confidentiality is very important as it promotes confidence between the patient and the doctor. Patients are most likely to provide accurate information when they are not worried about public exposure.

Patients were attended to in the clinics on a first-come-first-serve basis and 86% found the waiting area of the hospital site to be both suitable and comfortable. The reason attributed to this high rate of suitability is the fact that most of the ART site structures were either renovated or donated by NGOs via the USA President’s Emergency Plan for AIDS Relief (PEPFAR) funds.

The majority of the patients surveyed indicated that they had to wait for at least one hour before being attended to. Human resource shortages such as doctors and nurses were a factor highlighted by the patients who complained about long waiting times at the clinics. This result is comparable to a previous study carried out by Wouters *et al.*^10^ which identified lack of human resources as being a causal factor for long waiting times.

### Communication

The majority of the patients reported that they were, in general, satisfied regarding communication with the attending healthcare provider. Almost all patients (98%) interviewed responded that their healthcare providers were polite to them during their consultation. These results are similar to another South African study by Magoro *et al.*^17^ where patients reported that doctors and nurses were kind, polite, respectful, and attentive during consultations. A patient satisfaction survey conducted in Singapore by Molina *et al.*^18^ reported that the courtesy and respect displayed by doctors has a positive impact on patient satisfaction. Also, patients are more likely to miss appointments or to fail to follow medical advice when poor communication exists.^18^

### Medicine management

All but three patients across all the ART hospital sites received their medication on time every month at the pharmacy with few or no complaints. The results of this study agree with a similar study carried out by Karunamoorthi *et al.* in Addis Ababa, where 73% of the patients were very satisfied with the pharmacy service.^19^

Eighty-five percent of the patients collected their TB and/or other chronic medication on the same day at the same pharmacy, as the services at these sites are integrated. This result implied that patients would not have to come to the clinic twice which saved them time and money, an important consideration in their socio-economic background.

An additional factor that could also be linked to the patient satisfaction at ART hospital sites is that the majority of patients (98%) understood the directions on the labels clearly, which implied that they knew how to take their medication. This might be linked to the high literacy levels of the patients. From the individual questions, patients from all the ART sites showed adequate knowledge regarding all their treatment regimen categories. The patients described the exact quantities and frequency of usage of their treatment regimens. The high literacy level of the patients and clear directions on the medicine labels may be responsible for this trend.

The majority of the patients (81%) preferred to collect their medicines from the main pharmacy rather than a pharmacy that is separated from the general outpatient pharmacy.

### Satisfaction or dissatisfaction with care

The overall satisfaction of patients included in this study was very high (98%), as is seen with respect to the general ART care provided by the facilities. This result is similar to that obtained in a study of an ART programme in the Free State.^10^

A study carried out by Chimbindi, Bärnighausen and Newell^11^ on patient satisfaction with HIV treatment and TB care in rural KwaZulu-Natal, South Africa showed that overall patient satisfaction was high but patients expressed some dissatisfaction with certain dimensions of the quality of care, including an inability to talk to health workers about their treatment and problems, time spent in queues waiting to be examined and facility cleanliness.

In this study, although 95% of the patients found their ART hospital site to be convenient and were happy with the services they received at the ART hospital sites, 78% experienced transport problems and 61% indicated that they would rather transfer to a facility closer to their home.

The reasons why seven (2%) patients indicated dissatisfaction with services included human resource shortages (especially doctors and nurses), bad staff attitude (such as impoliteness on the part of the nurses), non-protection of their confidentiality and dirty toilets.

### Limitations to the study

The fact that participants in this study may have answered questions to please the interviewer is acknowledged. It is also acknowledged that at another time or under other circumstances, the outcomes may be different. Although proportional random sampling was used to determine the number of patients at each ART hospital site, convenience sampling was used to select the required number of patients at the ART hospital site and the sample might not be representative.

## Conclusion

The findings of this study indicated general high satisfaction of patients with the ART-related services at the accredited ART sites in the Gert Sibande District. The major factors contributing to satisfaction included the availability of medicines, knowledge regarding how to take their medication and general satisfaction with providers. The major factors contributing to dissatisfaction (mentioned by seven respondents) included waiting too long, confidentiality issues, shortage of staff and dirty toilets.

Despite many efforts and successes with satisfaction measurement, evidence shows that more work in this area is still needed. One of the primary challenges has been in sustaining patient/customer satisfaction improvement initiatives in the face of competing priorities and diminishing resources. Patient satisfaction is important if service improvement is to be translated into outcomes meaningful to patients, especially improved quality of life.

It is therefore recommended that the results of this study be analysed and incorporated into the service planning process of the department and the departmental policy makers integrate the learning opportunities from patient feedback into their quality improvement plans. This, together with a successful down-referral system where a large population of stable ART patients at treatment-initiation sites could be down-referred, will increase capacity and will also reduce long waiting times and save the patient money. A regular monitoring and evaluation plan is also recommended.
